# The relationship between BMI and depression: a cross-sectional study

**DOI:** 10.3389/fpsyt.2024.1410782

**Published:** 2024-10-22

**Authors:** Hongyu Cui, Ying Xiong, Chengmin Wang, Jiaming Ye, Weisen Zhao

**Affiliations:** ^1^ Department of Psychiatry, the First Affiliated Hospital of Harbin Medical University, Harbin, Heilongjiang, China; ^2^ Department of Dermatology and Venereology, Shenzhen Longgang Center for Chronic Disease Control, Shenzhen, Guangdong, China; ^3^ Department of Mental Health, Shenzhen Longgang Center for Chronic Disease Control, Shenzhen, Guangdong, China; ^4^ Department of Biobank, Suzhou Center for Disease Control and Prevention, Suzhou, Jiangsuu, China

**Keywords:** depression, body mass index, relationship, restricted cubic spline, subgroup analysis, risk factor

## Abstract

**Introduction:**

Mental health problems, especially depressive symptoms, are becoming increasingly prominent, posing a significant risk to public health. Changes in the body mass index (BMI) may impact an individual’s mental health, however, the relationship between BMI and depressive symptoms is unclear. The purpose of this study was to investigate the association between BMI and depressive symptoms.

**Methods:**

Using a multi-stage sampling method, 10,686 adults in Longgang District, Shenzhen City, Guangdong Province, China, were selected for participation in this study. Surveys were distributed in 2020 and 2021 to measure participant demographic data and health. Binary logistic regression, restricted cubic spline regression, and subgroup analyses were performed to explore the relationship between BMI and depressive symptoms.

**Results:**

The results showed a U-shaped relationship between BMI and depression. Both obesity and underweight increased the risk of depression among the participants, especially in subgroups of participants who were young, highly educated, single and employed.

**Conclusion:**

These findings suggest that adults should try to maintain a normal body weight as a way to prevent depression and maintain their physical and mental health.

## 1 Introduction

Depressive disorder is a pervasive mental disorder characterized by a prolonged and constant sense of melancholy or diminished interest in activities. It is closely associated with suicidal behaviours (1). Globally, an estimated 280 million individuals suffer from depression (2), with more than 50 million in China (3). However, China lacks mental health resources, and the prevalence of depression among Chinese adults is currently about 6.9%, higher than the global prevalence (about 5%) (2, 4). Depression is the tenth leading cause of all-cause disability-adjusted life years (DALYs) in China (5), but its complex pathogenesis has not yet been clarified and many risk factors remain to be explored.

To date, several studies have suggested that there may be an association between overweight (or obesity) and depression (6–10). This is supported by mechanism studies. For example, obesity-related inflammatory processes can affect the occurrence and development of depression (11, 12). There is also evidence indicating that weight perceptions influence mental well-being (13). The prevalence of obesity has doubled globally in the past 30 years, with over 2.5 billion adults being classified as overweight in 2022, including 890 million who were considered obese, while 390 million individuals were considered to be underweight (14). According to Chinese standards, between 2015 and 2019, 34.3% of individuals were considered overweight, while the rate of obesity was 16.4% (15). Given the increasing prevalence of abnormal weight, it is particularly important to explore the relationship between body mass index (BMI) and depression. However, the majority of existing research that has examined the association between weight and depression has focused on overweight (or obesity) and depression, with a predominant emphasis on specific demographic groups such as women, children and the elderly, rather than the broader general population.

Shenzhen, a mega-city in China, is one of the pilot cities for the development of a Psychosocial Service System. Accordingly, there is significant concern about the mental health of the general public in this city. A cross-sectional survey found that the prevalence of all types of mental illness among adults in Shenzhen was as high as 21.87% (16). Further, the prevalence of depressive disorders was 7.74% (17), which is higher than the national level. This indicates that Shenzhen faces a significant challenge in preventing and intervening in depression.

Therefore, the aim of this study was to explore the association between BMI and depression among adults in Shenzhen, with the goal of identifying key subgroups that necessitate focused attention. The ultimate goal of this study was to establish a scientific foundation for future prevention and control strategies for depression, and enhanced the treatment and management approaches for affected patients.

## 2 Materials and methods

### 2.1 Sample and sampling

A cross-sectional study in 2005 showed that the prevalence of depression among adults in Shenzhen was 7.74% (17). However, this estimate was established 15 years ago, and rapid social change may have brought about a general increase in psychological pressure and stress (18). Further, the current study was conducted during the COVID-19 pandemic, where there was undoubtedly an exacerbation of psychological pressure. Thus, we decided to take 10% as an estimate of the prevalence of depression. Estimation of the required sample size for this study was performed using the Exact (Clopper-Pearson) Formula for Calculating Confidence Intervals for One Proportion in PASS 2021 software. The significance level α was set at 0.01 and the quantity δ for permissible error was set at 0.01. The required sample size was calculated to be 6071. Assuming a sample loss of 20%, the estimated required sample size was 7589.

Sampling for this study was conducted in 2020 and 2021 using a multi-stage sampling strategy. In the first stage, the Longgang District of Shenzhen was chosen as the sampling area based on the accessibility of this district. In stage two, two sub-districts were selected from among the 11 sub-districts in Longgang district using the simple random sampling method. In stage three, within each of the selected sub-districts, four communities were selected using the population size ranking systematic sampling method (eight communities were selected in total). In stage four, in each selected community, four resident groups were selected by the simple random sampling method (a total of 32 resident groups were selected). In stage five, within each of the selected resident groups, 240 households were selected using the simple random sampling method; all usual residents aged 18 years and above in the selected households were investigated (a total of 7,680 households were selected). The inclusion criteria for participation were: aged 18 years or above; has resided continuously in Shenzhen City for more than six months; is able to complete the survey independently or with assistance from others. A total of 10,686 surveys were distributed. Surveys with missing or abnormal values for height and weight, and those with excessively short response times, incomplete data and obvious logical errors, were excluded. Finally, a total of 9,995 surveys were analysed from adult residents in Shenzhen City. All participants provided informed consent before participation. This study was performed in accordance with the Declaration of Helsinki.

### 2.2 Methods

A self-administered survey was used to collect information about the participants, including age, gender, education, marital status, economic status, employment status, height, weight, smoking and drinking. Further, the severity of depressive symptoms was assessed by the Chinese version of the Patient Health Questionnaire-9 (PHQ-9). The items in the scale cover nine symptom criteria for the diagnosis of clinical depression according to the Diagnostic and Statistical Manual of Mental Disorders Fourth Edition (DSM-IV) and have shown good reliability and validity (19). In a cross-sectional study of depression in China, the PHQ-9 demonstrated good reliability and validity. The Cronbach α coefficient of the PHQ-9 was 0.839. Taking the results of the Mini-International Neuropsychiatric Interview as the gold standard, the area under the receiver operating characteristic (ROC) curve of the PHQ-9 results for all subjects was 0.898 (20). Each item in the PHQ-9 is scored as follows: 0 = not at all, 1 = several days, 2 = more than half the days, and 3 = nearly every day. The total score ranges from 0 to 27, with a higher score indicating more severe depressive symptoms. Total scores of 0-4, 5-9, 10-14, 15-19 and 20-27 indicate no, mild, moderate, moderately severe and severe depression, respectively. In this study, a score ≥ 5 was considered to indicate the presence of depression (21). BMI was calculated according to the guidelines for the prevention and control of overweight and obesity in Chinese adults, with BMI < 18.5 kg/m^2^, 18.5-24.0 kg/m^2^, 24.0-28.0 kg/m^2^ and ≥ 28.0 kg/m^2^ defined as underweight, normal weight, overweight, and obesity, respectively (15).

### 2.3 Quality control

Quality control before the study. Consult with relevant experts to design the electronic questionnaire. Reminders were set up in the survey to ensure that the participants submitted the complete questionnaire. Centralized and unified training was provided for the investigators.

Quality control during the survey. The investigators had to adopt uniform investigation standards, carefully check the surveys obtained, record the study procedures, and treat the participants politely.

Quality control after survey. Convert the electronic questionnaire into a data format. The data were double-checked to remove participants who completed the survey in too little time and who had obvious logical errors.

### 2.4 Statistical analysis

Statistical analysis and visualization were performed using R 4.3.2 software. Statistical significance was set at 0.05. Normally distributed data were described by $\bar{x}\;\pm \;s$ , and differences between groups were examined by ANOVA. Non-normally distributed data were described by *M* (*Q*_1_, *Q*_3_), and differences between groups were examined using the rank sum test. Categorical data were described by frequencies (constitutive ratios), and differences between groups were examined by the χ^2^ test. The association between BMI and depressive symptoms was analysed using a binary logistic regression model. The dose-response relationship between BMI and depressive symptoms was explored using the restricted cubic spline (RCS) model. Subgroup analyses were also performed to investigate the association between BMI and depressive symptoms.

## 3 Results

### 3.1 Demographic data

The average age of the participants was 34.0 (29.0, 44.0) years, among which, the average age of individuals with depressive symptoms was 32.0 (27.0, 40.0) years. The percentage of participants with depressive symptoms was 11.8% (1,176). Overall, 62.4% (6,240) of participants were female, and 49.4% (4,942) had an education level of college or higher. The average BMI of the sample was 22.10(20.08, 24.22) kg/m^3^.

There were statistically significant differences in the prevalence of depression among residents grouped by age, education, marital status, family economic status, employment status, smoking, drinking, and BMI subgroups, as shown in **Table 1**.

**Table 1:** Demographic information of participants.

	Overall	Depression	*P*
Age [M (Q1, Q3)]	34.00 (29.00, 44.00)	32.00 (27.00, 40.00)	<0.001*
BMI level [M (Q1, Q3)]	22.10 (20.08, 24.22)	21.85 (19.57, 24.22)	0.002*
Gender (%)			0.882
Male	3755 (37.57%)	439(11.69%)	
Female	6240 (62.43%)	737(11.81%)	
Education (%)			<0.001*
Junior high school and below	2477 (24.78%)	230(9.29%)	
High school	2576 (25.77%)	269(10.44%)	
College and above	4942 (49.44%)	677(13.70%)	
Marital status (%)			<0.001*
Married	8182 (81.86%)	754(9.22%)	
Unmarried/divorced/widowed	1813 (18.14%)	422(23.28%)	
Family economic status (%)			<0.001*
Better	1172 (11.73%)	139(11.86%)	
Ordinary	8031 (80.35%)	814(10.14%)	
Poor	792 (7.92%)	223(28.16%)	
Employment status (%)			<0.001*
Be in employment	8370 (83.74%)	966(11.54%)	
Unemployed/retired	1625 (16.26%)	210(12.92%)	
Smoking (%)			<0.001*
Yes	1451 (14.52%)	249(17.16%)	
No	8544 (85.48%)	927(10.85%)	
Drinking (%)			<0.001*
Yes	1362 (13.63%)	256(18.80%)	
No	8633 (86.37%)	920(10.66%)	
BMI subgroups (%)			<0.001*
Low weight	930 (9.30%)	167(17.96%)	
Normal	6225 (62.28%)	690(11.08%)	
Overweight	2373 (23.74%)	252(10.62%)	
Obese	467 (4.67%)	67(14.35%)	
Total (%)	9995 (100.00%)	1176(11.77%)	

*Statistically significant.

### 3.2 Binary logistic regression

After adjusting for age, sex, education, marital status, economic status, employment status, smoking, and drinking, binary logistic regression showed a no linear association between BMI and the risk of depressive symptoms (*P* > 0.05). Compared with the normal BMI group, the obese and underweight groups had higher risks of depressive symptoms (*P* < 0.05) (**Table 2**).

**Table 2:** Binary logistic regression of BMI and depressive symptoms.

	Model1[*OR(95%CI)*]	Model2[*OR(95%CI*)]	Model3[*OR(95%CI)*]
BMI subgroups			
Low weight	1.756 (1.459~2.113)*	1.577 (1.305~1.905)*	1.350 (1.107~1.647)*
Normal	1	1	1
Overweight	0.953 (0.818~1.110)	1.016 (0.870~1.187)	1.048 (0.893~1.230)
Obese	1.344 (1.025~1.761)*	1.384 (1.055~1.817)*	1.343 (1.013~1.782)*
BMI level	0.976 (0.957~0.996)*	0.994 (0.974~1.015)	1.005 (0.984~1.026)

*Statistically significant.

Model 1: no covariates were adjusted.

Model 2: adjust for: age (smooth); gender.

Model 3: adjust for: age (smooth); gender; education; marital status; economic status; employ-ment status; smoking; drinking.

### 3.3 RCS analysis

**Figure 1:**
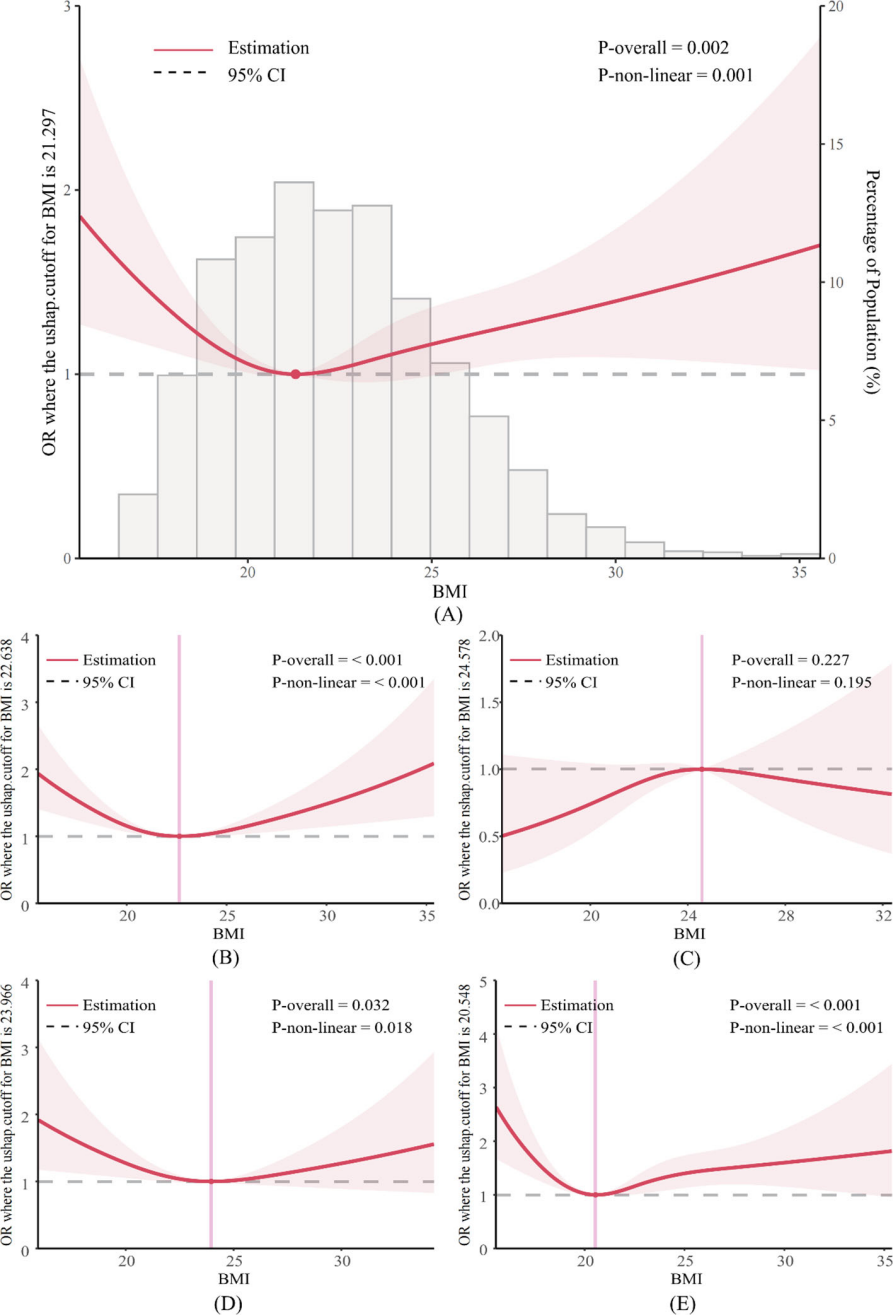
RCS curve of BMI levels and risk of prevalence of depression. **(A)** in total study population; **(B)** in <45 years old; **(C)** in ≥45 years old; **(D)** in male; **(E)** in female.

The RCS analysis revealed a non-linear relationship between BMI and depressive symptoms. That is, a U-shaped association was observed, where the depression OR was the lowest when BMI was 21.3 kg/m^3^. A U-shaped relationship was also observed for the various investigated subgroups, including those under the age of 45, and males and females, respectively, where the lowest ORs for depressive symptoms corresponded to a BMI of 22.6 kg/m^3^, 24.0 kg/m^3^and 20.5 kg/m^3^, respectively. However, these nonlinear associations were not observed in people aged 45 years and older (**Figure 1**).

### 3.4 Subgroup analysis

**Figure 2:**
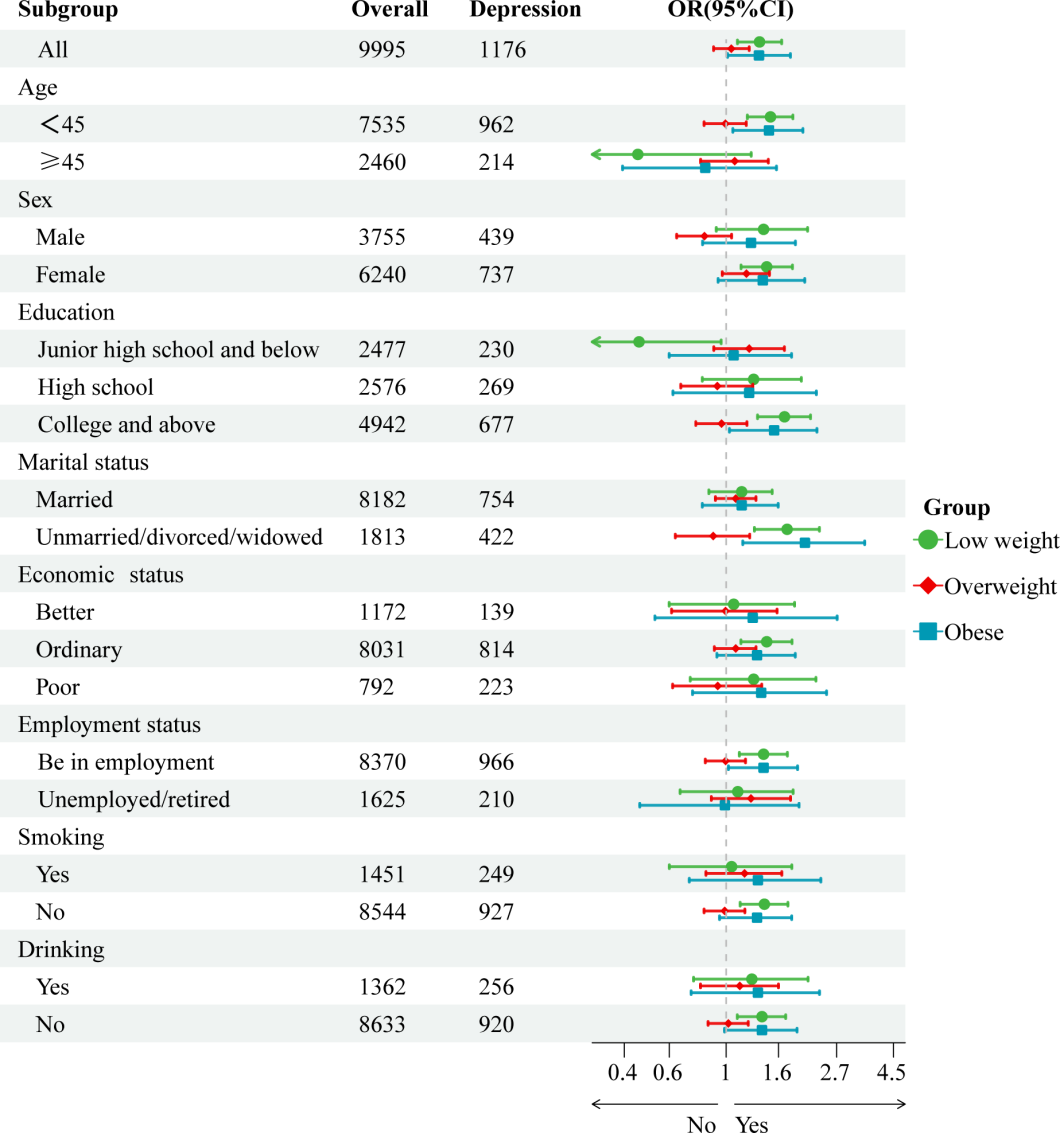
Forest plot of association between BMI and depressive symptoms in different demographic subgroups.

Normal BMI was used as a reference, and both underweight and obesity were associated with the risk of depression. Underweight increased the risk of depression in people below the age of 45, in females, in individuals with a junior high school and below education, in individuals with a college and above education, in unmarried/divorced/widowed individuals, in individuals with an ordinary economic status, in employed individuals, and in non-smokers and non-drinkers. Obesity increased the risk of depression in people below the age of 45 years, those with a college and above education, those who were unmarried/divorced/widowed, and those who were employed (**Table 3**, **Figure 2**).

**Table 3:** Binary logistic regression analysis of BMI and depression in different demographic subgroups.

Group	Low weight [*OR*(95%*CI*)]	Normal	Overweight [*OR*(95%*CI*)]	Obese [*OR*(95%*CI*)]
Age				
<45	1.490 (1.210~1.822)*	1	0.993 (0.820~1.198)	1.470 (1.062~1.994)*
≥ 45	0.452 (0.109~1.252)	1	1.080 (0.795~1.461)	0.829 (0.394~1.572)
Gender				
Male	1.400 (0.915~2.079)	1	0.823 (0.641~1.050)	1.250 (0.809~1.864)
Female	1.440 (1.143~1.815)*	1	1.200 (0.966~1.475)	1.390 (0.930~2.026)
Education				
Junior high school and below	0.457 (0.186~0.956)*	1	1.230 (0.895~1.688)	1.070 (0.599~1.800)
High school	1.280 (0.807~1.966)	1	0.924 (0.665~1.271)	1.230 (0.620~2.249)
College and above	1.690 (1.326~2.135)*	1	0.960 (0.761~1.205)	1.540 (1.030~2.261)*
Marriage				
Married/coupled	1.150 (0.856~1.512)	1	1.090 (0.908~1.307)	1.150 (0.807~1.596)
Unmarried/divorced/widowed	1.730 (1.289~2.313)*	1	0.890 (0.633~1.236)	2.030 (1.161~3.474)*
Economic status				
Better	1.070 (0.599~1.850)	1	0.995 (0.612~1.580)	1.270 (0.527~2.712)
Ordinary	1.440 (1.142~1.808)*	1	1.090 (0.899~1.308)	1.320 (0.920~1.861)
Poor	1.280 (0.724~2.241)	1	0.927 (0.618~1.377)	1.370 (0.739~2.466)
Employment status				
Be in employment	1.400 (1.126~1.734)*	1	0.995 (0.829~1.190)	1.400 (1.021~1.900)*
Unemployed/retired	1.110 (0.661~1.825)	1	1.250 (0.875~1.784)	0.989 (0.460~1.926)
Smoking				
Yes	1.050 (0.600~1.804)	1	1.180 (0.834~1.649)	1.330 (0.718~2.342)
No	1.410 (1.134~1.742)*	1	0.987 (0.820~1.183)	1.320 (0.942~1.803)
Drinking				
Yes	1.260 (0.746~2.088)	1	1.130 (0.794~1.600)	1.330 (0.730~2.314)
No	1.380 (1.105~1.708)*	1	1.020 (0.850~1.220)	1.380 (0.986~1.891)

*Statistically significant.

## 4 Discussion

This large-scale mental illness-related research project organized by the Longgang District of Shenzhen, found that 11.77% of adults in Shenzhen suffer from depression. This estimate is much higher than the global prevalence of depression among adults (approximately 5%) (2). Thus, mental health issues should be given more attention in this population. In particular, the prevalence of depressive symptoms was higher among those who were unmarried/divorced/widowed, unemployed/retired, smoked cigarettes and drank alcohol.

This study found a U-shaped association between BMI and the prevalence of depressive symptoms in adults. This suggests that being underweight or overweight increases the risk of depression, similar to the findings of previous studies (22–25). The mechanisms by which an unhealthy weight leads to depression are diverse. Many studies have shown that obese people have a higher risk of depression, and this may be related to increased inflammatory response (26), leptin resistance (27, 28), cortisol-binding receptor damage (29), and disordered intestinal flora (30). On the other hand, obesity and underweight, as unhealthy physical characteristics, may have negative effects through social interaction, body image and self-esteem, thus increasing the risk of depression (31, 32).

In contrast to the current findings, some studies have found a significant positive association between BMI and depression (33, 34). For example, a German cross-sectional study found that obese adults had a significantly higher risk of depression than non-obese individuals (35). It is possible that the non-obese subgroups (i.e., underweight, normal, and overweight subgroups) masked the effect of underweight on depression. In the above study, mediation analysis showed that the association between BMI and depressive symptoms was mediated by physical health status. Thus, the authors reported that the likely reason for the high prevalence of depression in obese individuals is that obesity causes changes in health status which, in turn, mediate the development of depression (35).

Similarly, in contrast to the current study, some studies have found an inverse association BMI and depression (36, 37). This may be because these studies targeted specific populations (only middle-aged and older adults), while our study targeted adults (including middle-aged and older adults). The “jolly fat” hypothesis (fatter people are happier) could be used to explain the results of a cross-sectional study of Chinese middle-aged and older adults, where a negative association between BMI and depression in middle-aged and older adults was observed (36, 38). This hypothesis may also explain why as BMI decreases, the risk of depression increases. That is, psychological stress caused by rapid weight loss, such as diet control, may also increase the risk of depression (36, 38).

The results of the RCS in our study revealed that the relationship between BMI and the prevalence of depressive symptoms was U-shaped association across the adult population. The lowest OR for depression was observed at a BMI of 21.3 kg/m^2^, which corresponds to the normal BMI range. Similarly, RCS analysis of different age subgroups showed a U-shaped relationship between BMI and the risk of depressive symptoms in young people, but not in middle-aged and older adults. This suggests that BMI is more strongly associated with depressive symptoms in younger populations. This is a novel finding. The U-shaped association was also observed in the gender subgroups. However, we observed something even more interesting. Specifically, the lowest risk of depression was found in males and females with BMI values of 24.0 kg/m^2^ and 20.5 kg/m^2^, respectively. Combined with the RCS curve morphology and trends in men and women it appears that the risk of depression in women is more likely to be affected by changes in BMI than in men, possibly because obesity or underweight causes an increased psychological burden in women. A study based on the 2005-2018 NHANES data showed that at the lowest risk of depression, women had a lower optimal BMI value (21.1 kg/m^2^) than men (25.2 kg/m^2^), which is consistent with our speculation (22).

Further subgroup analyses revealed that among those who were employed, both underweight and obesity increased the risk of depression. This may be due to the presence of more work and life stressors, and thus, an unhealthy BMI may pose a greater mental burden (39). Further, among those who were unmarried/divorced/widowed, both underweight and obesity increased the risk of depressive symptoms. This may be related to the experience of loneliness among this population, which can increase the risk of mental illness (40). Unexpectedly, among smokers and drinkers, abnormal BMI was not associated with depressive symptoms. This may be because smoking and drinking increase a sense of pleasure and reduce mental stress, thus alleviating the onset of depressive symptoms (41).

The strengths of this study include the large sample size and examination of the adult population in general, as compared to studies that only included the elderly or young people. Thus, this study offers more a comprehensive insight into the association between BMI and depression among adults. Further, RCS analysis was conducted to explore the relationship between BMI and the risk of depression in the total sample as well as in different subgroups, which is rarely performed in the available literature. Nonetheless, this study still has some limitations. First, this was a cross-sectional study and as such, causal inferences cannot be made. Cohort studies are needed to investigate these causal relationships in the future. Second, the data in this study were based on self-report. Thus, inaccuracies and information bias cannot be excluded. In addition, important influencing factors, such as physical fitness, activity status and diet, were not adjusted for in this study and may have confounded the results. Future mediation studies of the relationship between BMI and depression may help to elucidate the potential causal mechanisms.

## 5 Conclusions

These findings reveal a U-shaped association between BMI and the risk of developing depression. That is, being underweight or obese was found to increase the risk of developing depression in adults. Importantly, a U-shaped association between BMI and depression was observed in the younger subgroup but not in the middle-aged and older subgroups. The risk of depression in the female population was more likely to be associated with a change in the BMI, as compared to male population. Moreover, high rates of depression were observed among smokers and drinkers, but an unhealthy BMI was not associated with an increased risk of depression in these groups.

## Data Availability

The raw data supporting the conclusions of this article will be made available by the authors, without undue reservation.
